# Supplementary material for: Structural Variations in Biobased Polyfurfuryl Alcohol Induced by Polymerization in Water
Source: Polymers (Basel). 2023 Mar 31;15(7):1745. doi: 10.3390/polym15071745 (PMC10096809; doi:10.3390/polym15071745)
Supplement: Supplementary file 1 [file polymers-15-01745-s001.zip › polymers-2274234-supplementary.pdf]

Table S1. Data obtained from the C=O quantification, enthalpies from DSC and the calculated conversion degrees.

C=O content	ΔH (J/g)	Conversion degree
0.19 ± 0.05	610	0.00
1.45 ± 0.17	470	0.23
1.75 ± 0.15	275	0.55
2.00 ± 0.18	145	0.76

Figure S1. Magnified HSQC spectra of PFA+ (blue) and PFA° (red) of the CH_3 area. The blue spectrum is stacked on top of the red one.

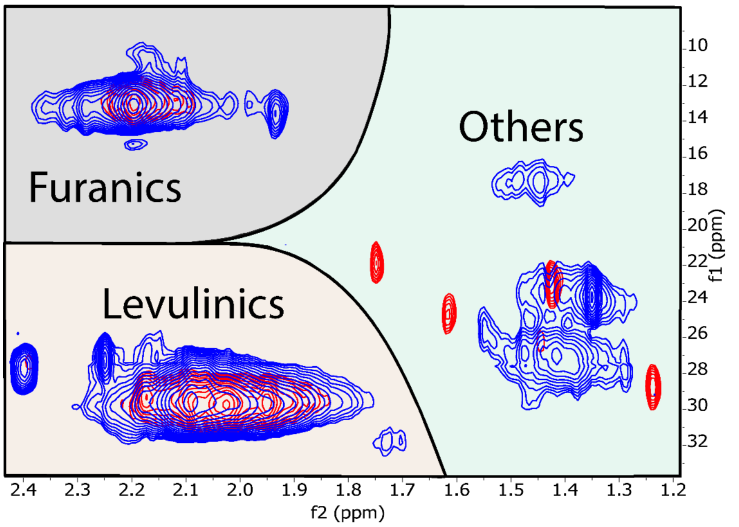

Figure S2. HMBC spectra of PFA° (red) and PFA+ (blue) focusing on the methyls. The red spectrum is on top of the blue one

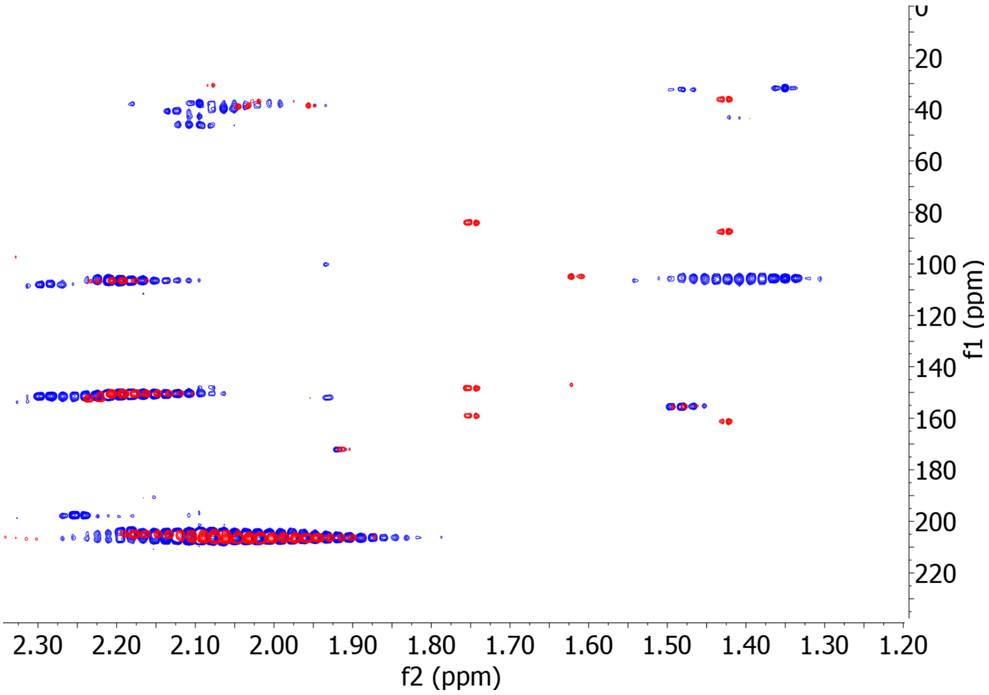

Figure S3. Magnified HSQC spectra of PFA+ (blue) and PFA° (red) of the CH_2 area. The blue spectrum is stacked on top of the red one

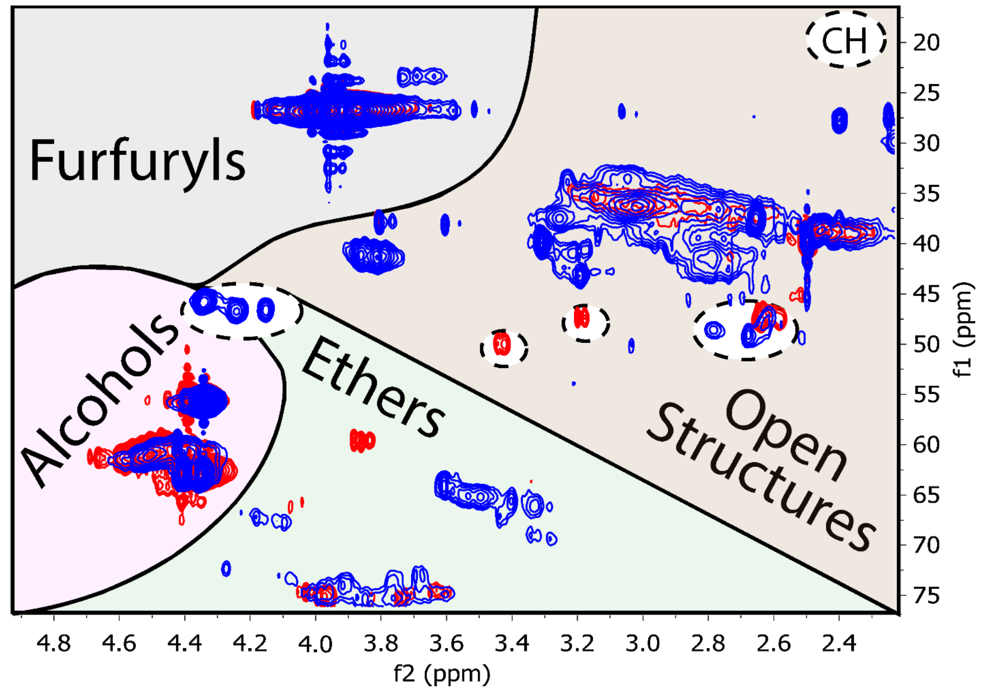

Figure S4. Magnified HSQC spectra of PFA+ (blue) and PFA° (red) of the dienes and ethers areas. The blue spectrum is stacked on top of the red one

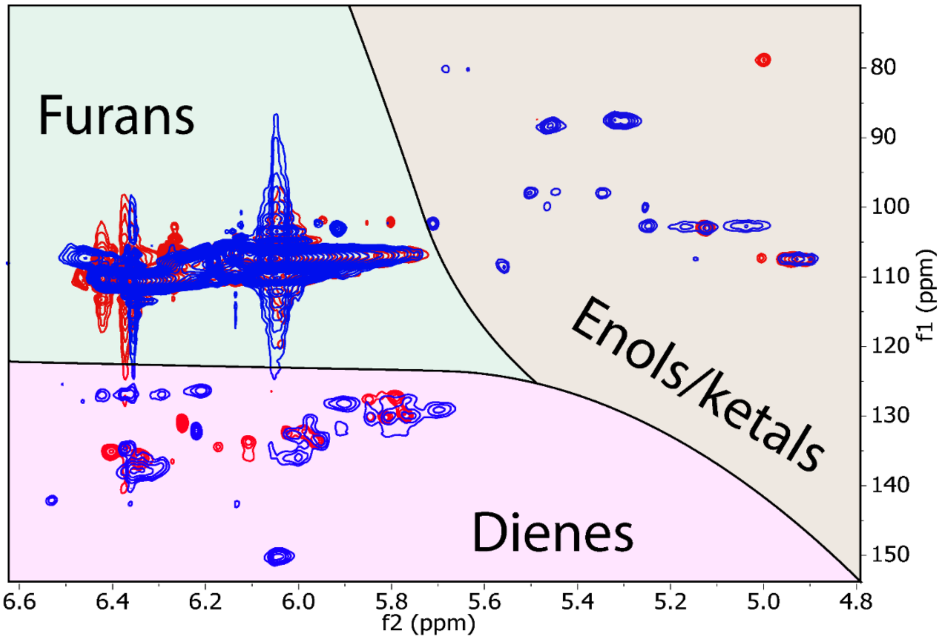

Figure S5. Magnified HSQC spectra of PFA+ (blue) and PFA° (red) of the aldehyde area. The blue spectrum is stacked on top of the red one

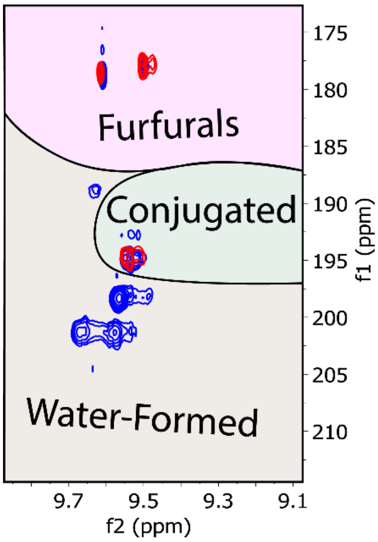

Table S2. ^{1}H and ^{13}C NMR predicted chemical shift of a furfuryl lactone unit

N°	δ (1H, ppm)	δ (^{13}C, ppm)
1	6.04	110.1
2	/	153.4
3	3.35	29.2
4	/	93.4
5	1.52	23.3
6	7.31	160.4
7	6.72	120
8	7	172.5

Table S3 Peak/structures assignments of the MALDI ToF spectra for PFA+ and PFA°

152 Da =

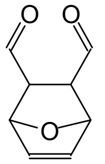

157 Da =

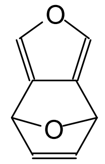

161 Da =

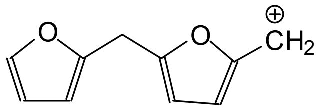

AND/OR

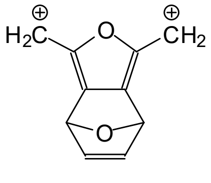

176 Da =

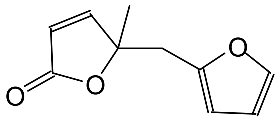

AND/OR

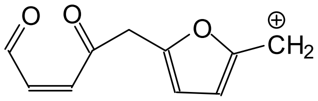

190 Da =

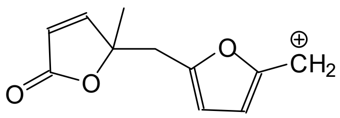

AND/OR

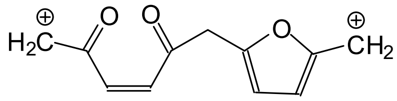

194 Da =

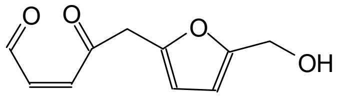

197-198 = no Na+, deprotonated

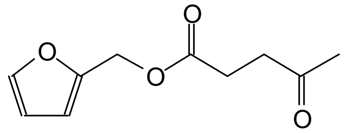

226 Da = no Na+, deprotonated

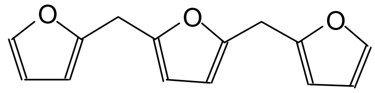

240-241Da = no Na+, more present in PFA+

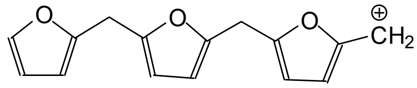

257 Da = protonated no Na+ (in PFA+), and **281Da, with Na+** in both PFA+ and PFA°. But more pronounced in PFA+ than in PFA°

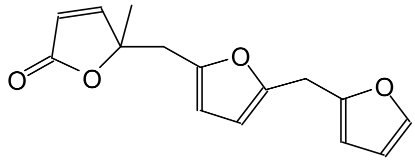

308 Da = no Na+

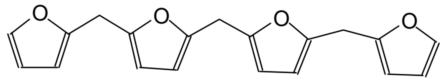

310 Da =no Na+, calc.310 Da , **and 334-335 Da**, with Na+

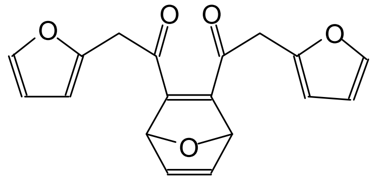

318.6 Da = with Na+, protonated

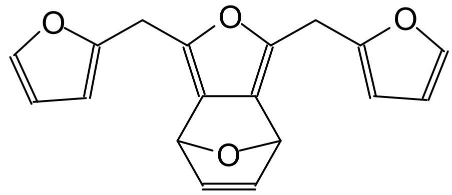

326-327 Da =no Na+ (both in PFA° and PFA+), and 350 Da with Na+ on in PFA° (small).Example of open structure of polyfuranics

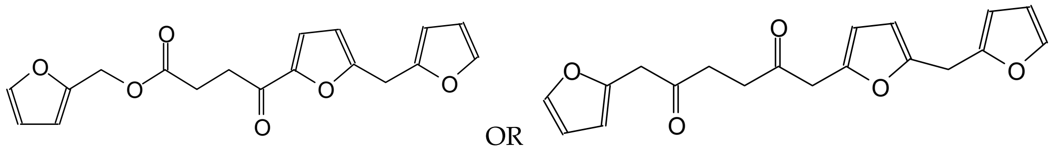

335 Da = with Na+, calc 321 Da, showing more open forms in PFA+

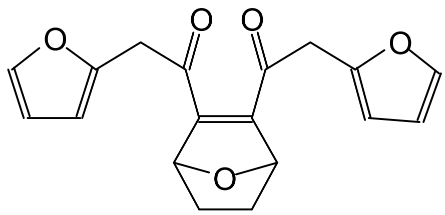

336-338 Da = no Na+, Calc. 337 deprotonated, 338 Da normal more present in PFA+, and **361 Da with Na+**

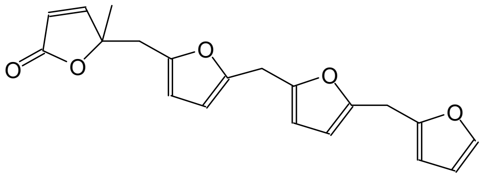

404 Da = no Na+, and 428 Da with Na+, and example of an end-chain conjugated aldehyde resulting from the oxidative ring-opening of furans in PFA resins. It is present on PFA+ but not really distinguishable in PFA°.

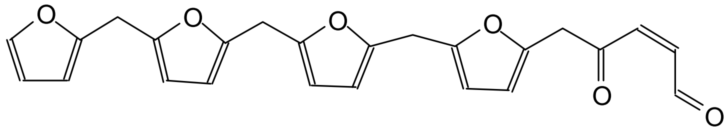

456 Da = no Na+, protonated, calc. 455 Da; **476-478 Da with Na+,** calculated 477 Da

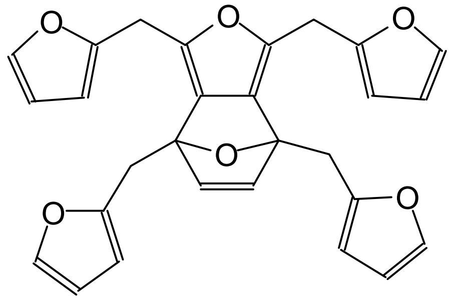

It shows that there are several more forms of this strcture in PFA+.
All the above indicates that the open ketones forms predominates even in the Diels Alder structures.

470 Da = no Na+, and 493 Da with Na+

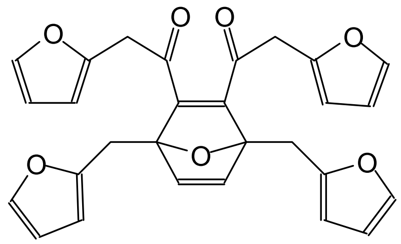

AND OR

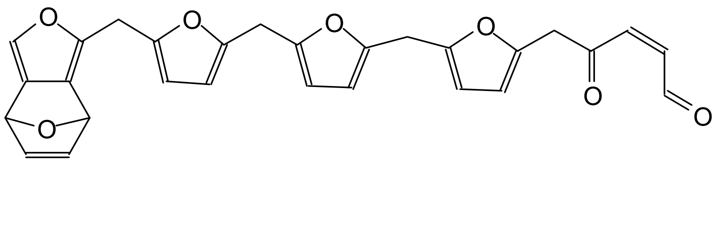

481-483 Da = no Na+, in PFA°

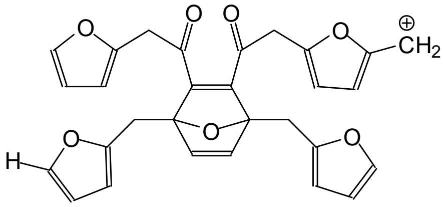

496 Da = no Na+ in both PFA° and PFA+, but more marked in PFA°

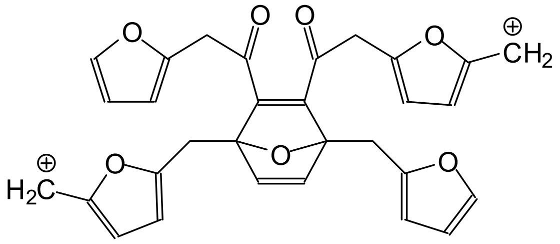

574-575 Da = with Na+,

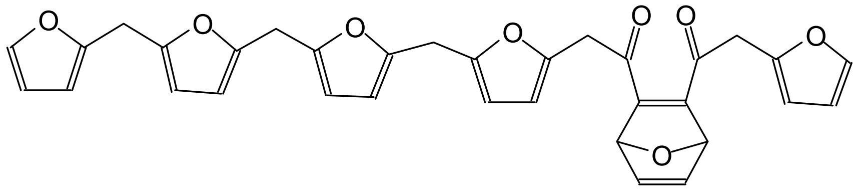

AND /OR

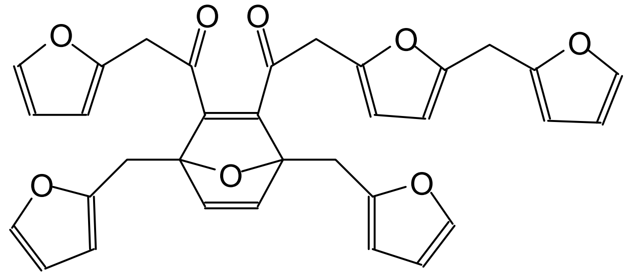

It is unnecessary to calculate and assigned higher oligomers structures as several isomer structures become possible.
